# A systematic review of lifestyle interventions for chronic diseases in rural communities

**DOI:** 10.21663/jgpha.5.404

**Published:** 2016

**Authors:** Selina A. Smith, Benjamin Ansa

**Affiliations:** 1 Institute of Public and Preventive Health, Augusta University, Augusta, GA; 2 Department of Family Medicine, Medical College of Georgia, Augusta University, Augusta, GA

**Keywords:** lifestyle intervention, dietary intake, physical activity, chronic disease, rural population

## Abstract

**Background:**

Rural Americans suffer disproportionately from lifestyle-related chronic diseases (e.g., obesity, diabetes, hypertension, cardiovascular disease, and breast cancer). Interventions that consider the distinctive characteristics of rural communities (e.g., access to healthcare, income, and education) are needed. As an initial step in planning future research, we completed a systematic review of dietary intake and physical activity interventions targeting rural populations.

**Methods:**

Manuscripts focused on dietary intake and physical activity and published through March 15, 2016, were identified by use of PubMed and CINAHL databases and MeSH terms and keyword searches.

**Results:**

A total of 18 studies met the inclusion criteria. Six involved randomized controlled trials; 7 used quasi-experimental designs; 4 had a pre-/post-design; and 1 was an observational study. Eight studies were multi-site (or multi-county), and 3 focused on churches. Primary emphasis by racial/ethnic group included: African Americans (6); Whites (2); Hispanics (3); and two or more groups (7). Most studies (17) sampled adults; one included children. Two studies targeted families.

**Conclusions:**

Additional lifestyle intervention research is needed to identify effective approaches promoting healthy diet and exercise and chronic disease prevention in rural communities. Studies that include rigorous designs, adequate sample sizes, and generalizable results are needed to overcome the limitations of published studies.

## INTRODUCTION

According to the United States Department of Agriculture (USDA, 2016), 17.5% of people in Georgia live in rural areas. Rural communities have various poverty-related challenges that affect health and health outcomes (e.g., inadequate housing and transportation, communication issues, and limited education) (Drozek et al., 2014). Relative to the general population, rural Georgians experience higher incidences of disease and morbidity, increased mortality rates, and lower life expectancies. Financial burdens, labor-intensive work, social isolation, stress, and low accessibility to health care are determinants of health faced by rural residents (Bennett et al., 2013). Common among rural dwellers are limited access to health care, lack of health insurance, long distances traveled for routine checkups and screenings, fewer doctors and healthcare providers, and late stages of disease presentation.

Chronic diseases such as obesity, hypertension, cardiovascular disease, type 2 diabetes, and cancers are prevalent, costly, and preventable health problems. In 2012, almost half of all US adults (117 million people) had one or more chronic health condition, and one of four had two or more such conditions (Ward et al., 2014). Currently, the estimated prevalence of obesity for adults aged 18 years and older is 28.9% nationwide and 30.5% for the state of Georgia. Also, 9.3% (29.1 million) of Americans and 9.9% (734,800) of Georgians are diagnosed with diabetes; 29% (70 million) of Americans and 35% of Georgians have hypertension; and 4.2% of American adults and 4.1% of Georgians have coronary heart disease (Levi et al., 2015; Blackwell et al., 2014). The 2016 estimates for new cases of invasive breast cancer are 246,660 for the US and 6,260 for Georgia (American Cancer Society [ACS], 2016; McNamara et al., 2015).

**Figure 1:**
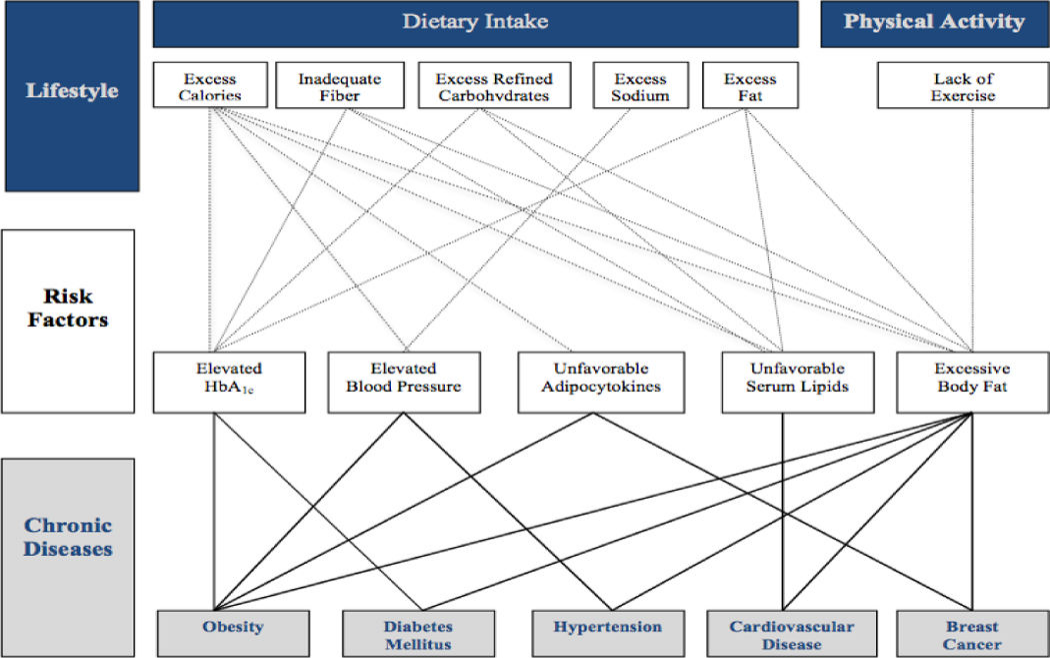
Lifestyle, risk factors, and chronic diseases

Many chronic diseases have associated lifestyle risk factors and are responsive to lifestyle modifications (Cecchini et al., 2010) (Figure 1). Lifestyle factors (e.g., excessive calories, refined carbohydrates, sodium, and fat; inadequate fiber; and lack of physical activity) as evidenced by risk indicators (e.g., elevated HgA_1c_ and blood pressure; unfavorable adipocytokines and serum lipids; and excessive body fat) are linked to chronic diseases (e.g., obesity, diabetes mellitus, hypertension, cardiovascular disease, and breast cancer).

Engaging in the recommended levels of physical activity and consuming a healthy diet can prevent several chronic diseases and reduce morbidity and mortality from these conditions (Willet et al., 2006). Adults with common chronic conditions who participate in comprehensive lifestyle modification programs experience rapid, significant, clinically meaningful, and sustainable improvements in biometric, laboratory, and psychosocial outcomes (Ricanati et al., 2011). Nearly 40% of all cancer deaths and 82% of cardiac deaths could be prevented and 91% of the cases of diabetes could be avoided through appropriate lifestyle changes, including adopting a simpler, healthier diet, and by following a consistent physical activity program (Aldana, 2005).

Although rural counties in the US have, relative to non-rural areas, higher rates of obesity, sedentary lifestyles, and associated chronic diseases, obesity and chronic diseases in rural communities have received little attention (Perri et al., 2008). As an initial step in planning future research, the authors of this report completed a systematic review of interventions related to dietary intake and physical activity targeting rural populations in the US. The goals were to examine the effectiveness of lifestyle interventions in rural communities and to determine salient features for replication in future studies.

## METHODS

**Figure 2:**
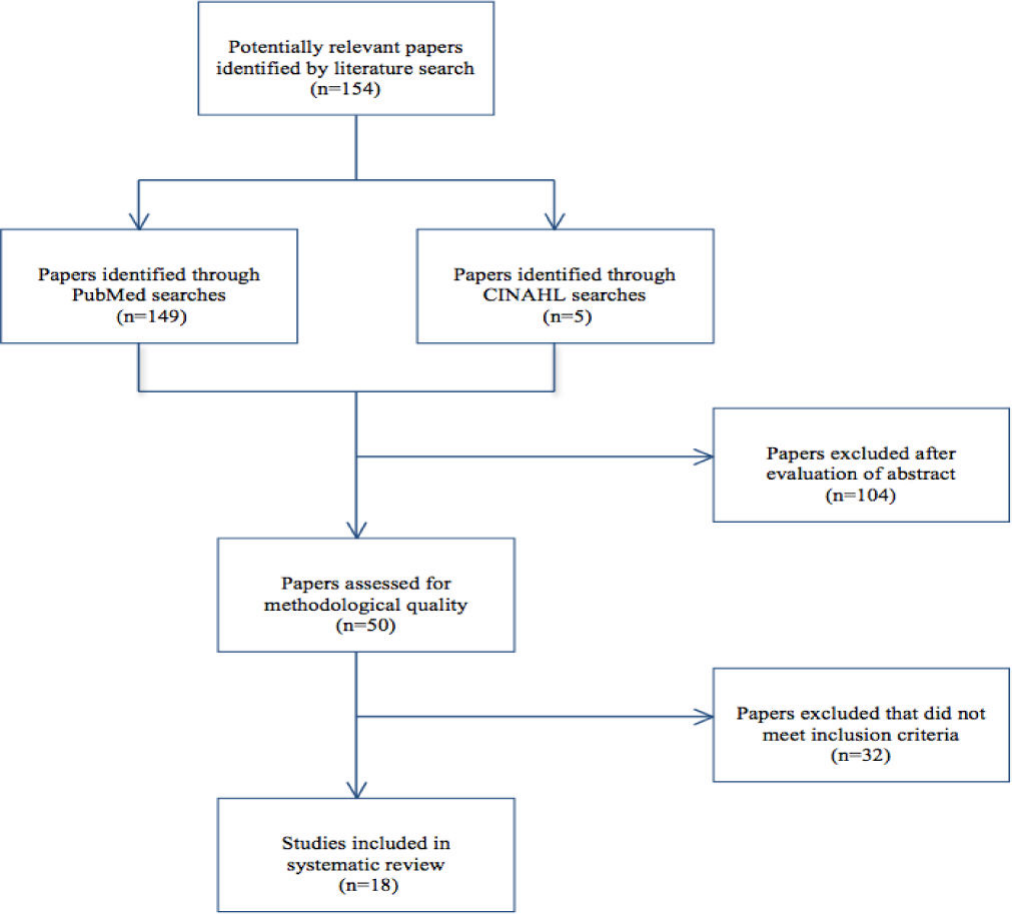
Flowchart of intervention selection process

The present review is based upon bibliographic searches of PubMed and CINAHL with relevant search terms. Articles published in English through March 15, 2016, were identified using the following MeSH search terms and Boolean algebra commands: (((dietary intake) or (diet) or (nutrition) AND (physical activity) or (exercise)) AND (rural)). Although the search criteria did not specify a begin date, the earliest article that met the search criteria was published in 2004. The searches were not limited to words appearing in the titles of articles. Information obtained from bibliographic searches (title and topic of article, information in abstract, geographic locality of a study, and key words) was used to determine whether to retain each article. In addition, reports included in Cochrane reviews (http://community.cochrane.org/cochrane-reviews) were identified. Only completed studies were included; study protocols were eliminated (Figure 2); 154 citations were found.

After screening the abstracts or full texts of these articles, 50 studies of rural populations were identified, all of which were conducted in the US. The inclusion criteria were completed studies of interventions focusing on dietary intake and/or physical activity and performed in rural areas in the US. Thirty-two reports that did not meet the inclusion criteria were not considered further. The two eligible studies identified in the CINAHL search overlapped with those identified in the PubMed search.

## RESULTS

A total of 18 studies met the search criteria (Table 1). Six included a randomized controlled approach; 7 used quasi-experimental designs; 4 had a pre-/post-design; and 1 was an observational study. Eight studies were multisite or multi-county, and 3 focused on churches or other institutions within rural communities. Primary emphasis by racial/ethnic group included: African Americans (6), Whites (2), Hispanics (3), and two or more groups (7). Most studies (17) focused on adults; 1 included children. Two studies targeted families.

Befort et al. (2012) conducted a quasi-experimental study with 35 White obese breast cancer survivors aged 46–74 years from three cancer centers in rural Kansas. The 6-month, group-based, weight control intervention incorporated self-regulation skills and social support to enhance changes in diet and physical activity. There were significant post-intervention changes for weight (>10% loss), fruit and vegetable consumption (+3.7 ± 4.3 servings/day), and physical activity (+1235 ± 832 kcal/week), and reductions in two biochemical mediators of breast cancer risk, fasting insulin (−16.7%) and leptin (−37.1%).

Brown et al. (2011) performed a pre-test, post-test control group study of 165 Mexican American men and women aged 35–70 living in a rural community on the Texas-Mexico border. The 6-month intervention compared culturally tailored diabetes self-management education (DSME) alone to DSME plus nurse case management (NCM). For both groups, positive changes in diet and physical activity and clinical outcomes were noted. Women had greater reductions in BMI relative to men. Participants with the most NCM contacts attended more DSME sessions, and higher attendance resulted in greater reductions in HbA_1c_ levels.

Carter et al. (2015) engaged 55 African American men and women in a year-long intervention in two rural Alabama counties (Bullock and Macon). For those in Macon County, physical activity included membership in Curves, floor exercise, or walking; those in Bullock County engaged in aerobics, dance, the gym, or walking. Individuals doing floor exercise lost the most weight (22.4 lb or 11.18% change) followed by those walking (6.1 lb or 3.40% change). For all physical activities, systolic and diastolic blood pressure decreased (−8.94 to −12.66 and −5.34 to −12.66 mm Hg, respectively).

Fahs et al. (2013) compared a stage-matched nursing and community intervention (SMN+CI) to CI alone in a 14-month, multisite, randomized control trial of 117 rural White, Black, and Hispanic women aged 35–65 years in rural counties in New York and Virginia. Significant increases in fruit and vegetable consumption and reduced diastolic blood pressure were noted in the SMN+CI cohort. The CI group had a significant reduction in total cholesterol.

In rural central Nebraska, Hageman et al. (2014) conducted a 12-month randomized controlled trial to reduce blood pressure among 289 women (primarily White), ages 40–69 with hypertension. Participants were randomized to standard advice, web-based intervention, or print intervention. For the web-based and print intervention cohorts, waist circumference, % calories from fat, saturated fat, servings of fruits and vegetables, and low fat dairy improved significantly. Improvements were observed in web-based vs. standard advice groups in systolic blood pressure (p=0.048) and estimated VO_2_max (p=0.037).

Focusing on diabetes self-management behaviors, Hu et al. (2014) completed a quasi-experimental, family-based study of 36 Hispanics (mostly women) and 37 members of their adult families. The 8-week pilot study, conducted in rural central North Carolina, consisted of two family sessions and eight weekly group sessions. Session content included education on diabetes self-management, exercise, food, and eating healthy. Among study participants, HbA_1c_ dropped by a mean of 0.41% from baseline to 1-month post-intervention.

In two rural counties in southwest Georgia, Kegler et al. (2012) conducted a coach-based, quasi-experimental intervention to improve food quality and physical activity in 90 households. Participating in the 6-week study were African American and White adults ages 40–70 years and their household members 18 years of age or older. Intervention households reported increased exercise relative to comparison households.

Kim et al. (2008) conducted a study of a faith-based weight loss intervention. With an intervention group and a delayed-intervention control group, a quasi-experimental design was used. The 73 participants (71% female, mean age 54.1 years, 100% African American) were from rural churches in North Carolina. Small groups led by trained community members met weekly for 8 weeks. The community members emphasized physical activity, healthy nutrition, and the connection of faith to health. The mean weight loss in the intervention group was 3.60 lb relative to 0.59 lb in the control group.

Landry et al. (2015) completed a 6-month, community-based, pre-post trial of an intervention consisting of motivational enhancement, social support, pedometer diary self-monitoring, and educational sessions. The participants were 269 adults (94% African American, 85% female, mean age 44 years) in Hattiesburg, Mississippi. The outcome measures were steps per day, fitness, dietary intake, and psychosocial construct measures. For the physical activity and dietary outcome variables, there were temporal changes only for steps per day and sugar intake. Sugar intake decreased by about 3 teaspoons, and physical activity increased by approximately 2,010 steps per day.

In a multi-site, cardiovascular disease prevention study, Lilly et al. (2014) investigated barriers to lifestyle change among 81 white, Hispanic, and African American women in Colorado, North Carolina, and West Virginia. Most participants (72%) reported significant post-intervention improvements in problem-solving skills (p<0.001), perceived stress (p<0.05), and maintenance of or increases in fruit and vegetable intake and physical activity.

Mayer-Davis et al. (2004) conducted a 12-month randomized clinical trial of 152 African American and White diabetic men and women in rural South Carolina. The study evaluated the effectiveness of a Diabetes Prevention Program-type intervention (intensive intervention), reimbursable-lifestyle intervention, and usual care (controls) on weight loss and HbA_1c_. Relative to controls, intensive-intervention participants had greater weight loss than controls (−2.6 kg vs. −0.4 kg, p<0.01). HbA_1c_ was reduced among all participants (p<0.05) but was not different between cohorts.

In three rural counties in South Carolina, Parker et al. (2010) conducted a church-based weight loss intervention among African American women. The study was developed with community involvement. The 35 participants, between the ages of 25 and 64 years, were not pregnant or breast-feeding. Two 10-week interventions (spiritually-based and non-spiritually-based) were pilot tested using a pre-post design. Physical activity was assessed using the Yale Physical Activity Survey. Both interventions led to significant reductions in BMI, but the spiritually-based intervention (z = −1.97, P<0.01) led to greater reductions. For the spiritually-based group, significant improvements were found for physical activity (*z* = −2.74, P<0.01).

In three rural North Carolina counties, Ries et al. (2014) conducted a project with a quasi-experimental design. The participants were 485 low-income, predominately minority women (63% African American) with a mean age of 47.5 years. The curriculum for the bi-weekly group meetings, held over a 6-month period, addressed physical activity, healthy eating, weight control, stress management, education, and job skills. For both African Americans (P<0.05) and whites (P<0.0001), intervention participants were more likely than comparison participants to move from contemplation to action/maintenance in regard to the goal of increasing physical activity. For all participants, progression in stages of change mediated the intervention effect on physical activity but not fruit and vegetable intake. Intervention group participants engaged in more minutes of physical activity per week (138 minutes) than comparison participants (86 minutes, P≤0.05).

Robles et al. (2014) conducted a 12-week prospective study of 33 children aged 8–11 years in Bailey County, Texas. The purpose was to assess the impact of a pharmacy-directed pilot study on dietary intake and physical activity during out-of-school time. Post-intervention changes were noted in BMI (−0.30, p<0.0001), waist circumference (−0.47, p<0.001), decreased consumption of fried/sweet foods, and increased exercise.

In Alabama’s “Black Belt,” Scarinci et al. (2014) conducted a community-based, cluster-randomized trial comparing two interventions: 1) promotion of physical activity and healthy eating (healthy lifestyle arm), and 2) promotion of breast and cervical cancer screening. Participants were 565 African American women of ages 45–65 years in six rural counties in Alabama. At the 12-month follow-up, participants in the healthy lifestyle arm showed significant positive changes (increased physical activity, increased fruit/vegetable intake, and decreased consumption of fried food). At 24 months, these positive changes were maintained for healthy eating behaviors but not for physical activity.

In a quasi-experimental, 6-month intervention related to diet and physical activity, Tussing-Humphreys et al. (2013) assigned eight churches to intervention or control. Conducted in the Lower Mississippi Delta Region, the study included 403 African American men and women with a mean age of 47 years. Adapted from the Body and Soul program (Resnicow et al., 2004), Delta Body and Soul included peer counseling, a focus on regional cuisine, a didactic physical activity session, and self-directed physical activity. In both study arms, there were significant increases for consumption of total fruits and vegetables (0.3[1.8] and 0.2[1.1]), aerobic physical activity (22%), and strength/flexibility (24%).

In the “Wellness for Women” clinical trial, Walker et al. (2009) randomly assigned 225 women from two similar rural areas to cohorts receiving a computer-tailored newsletter or a generic newsletter. The 12-month intervention and 12-month follow-up examined behavioral markers of activity and eating. Relative to the generic cohort, at 6 months and at 12 months, the ‘tailored’ group achieved greater strength (p=0.008 and p=0.002) and consumed a lower percent of calories from saturated fat (p=0.028 and p<0.001), respectively.

Zoellner et al. (2007) conducted a quasi-experimental study to evaluate a 6-month intervention focused on promoting physical activity and health through walking teams led by coaches, with self-monitoring and monthly 1-hour educational sessions. The participants were 83 rural residents in Hollandale, Mississippi (99% African American, 97% women). There were improvements in waist circumference (−1.4 inches), systolic blood pressure (−4.3 mmHg), and HDL-cholesterol (+7.9 mg/dL) (p<0.001). Self-reported walking per day was 44.8 (SD±52.2) minutes at enrollment and 65.9 (SD±89.7) minutes at 6 months (P=0.154).

## DISCUSSION

In the US, rural communities are a large, medically underserved group. Although rural populations in the US have higher rates of chronic diseases than non-rural areas (Befort et al., 2012; Patterson et al., 2004; Eberhardt et al., 2001), there are few interventions that address lifestyle risk factors among rural populations. We reviewed eighteen interventions targeting lifestyle that were implemented in rural areas in the US. Most of the interventions found, among study participants, significant positive changes in measurable outcomes, such as weight loss, increase in levels of physical activity, and consumption of fruits and vegetables and improvements in biochemical mediators. Only six of the studies used a randomized controlled approach, and only one, by Kegler et al. (2012), was conducted in rural Georgia.

Physical activity and a healthy diet continue to gain recognition as lifestyle interventions for use in primary and secondary prevention (Durstine et al., 2013; Warburton et al., 2006; Willett, 2002). Finding effective methods to reach and influence behavior change in residents of rural communities, who are at high risk for developing chronic diseases, is a public health challenge. In rural America, sociocultural and personal barriers, including the stress of living in poverty, low health literacy, and lack of experience and skill in accessing healthcare information services, remain as obstacles (Brown et al. 2011).

Most of the studies covered in this review employed strategies that were mindful of the socioeconomic barriers to healthcare delivery encountered by rural dwellers. The strategies included community-based participatory research (CBPR), distance-delivery methods (web-based, telephone, and mail), and case management.

CBPR, a framework through which evidence-based interventions (e.g., randomized trials) are developed and implemented in the context of community engagement, offers a partnership approach to research that equitably involves community members, organizational representatives, and researchers in all aspects of the research process (Scarinci et al., 2014). It enhances cultural appropriateness and encourages the building of trust between researchers and community members, thereby facilitating recruitment of participants. Carter et al. (2015), Kegler et al. (2012), Kim et al. (2008), Ries et al. (2014), and Scarinci et al. (2014) utilized the CBPR approach. Distance-delivery methods for implementing lifestyle modification interventions show promise for reaching rural women. The weight-loss intervention by Befort et al (2012) examined the effect of a group-based, weight control intervention delivered through conference call technology to obese breast cancer survivors living in remote rural locations. The intervention included weekly group phone sessions and a reduced-calorie diet incorporating prepackaged entrees and shakes. Physical activity gradually increased to 225 min/week of moderate intensity exercise. There were also significant changes for weight, diet, physical activity, serum biomarkers, and quality of life. Hageman et al. (2014) also utilized the distance-delivery method. Women in groups who had the intervention delivered by the web or print-mailed, improved more than the group receiving standard advice in regard to waist circumference, daily calories from fat, and daily servings of fruit and vegetables. Lifestyle modification interventions that incorporate web-based components offer advantages of providing tailored messages at low cost in order to reach a large audience across great distances, with convenience for the users. Case management is a strategy to coordinate healthcare services and provide more consistent levels of health care access. Brown et al. (2011) studied changes in diet and physical activity between an experimental group that was administered a diabetes self-management education (DSME) with access to a nurse case manager (NCM), and another group that had DSME only. Although there was non-significant improvement in diet and physical activity between the two groups, the number of NCM contacts was proportional to DSME attendance. Case managers, who help patients locate and manage resources, are advocates within the healthcare system who enhance communication among healthcare providers, patients, and their families (Brown et al. 2011).

The reviewed studies had various limitations. Most (11) had small sample sizes and uncertain generalizability; 5 lacked a control group; and 5 with control groups lacked randomization. Studies by Kegler et al. (2012), Lilly et al. (2014), and Robles et al. (2014) had short intervention periods. In some of the studies, self-reporting of dietary intake and physical activity were limitations.

For rural America and for Georgia in particular, there is paucity of evidence-based studies about lifestyle modification interventions. To achieve health equity between rural and urban communities, increased funding for research activities targeting lifestyle interventions in rural areas is needed. The promotion of more tailored interventions that increase physical activity and consumption of healthy diets among rural residents and that address the limitations of existing studies, is warranted.

## CONCLUSIONS

Relative to urban residents, rural residents are disadvantaged in terms of socioeconomic determinants of health, access to and availability of healthcare resources, and facilities that reduce lifestyle risk for chronic diseases. Consequently, among rural communities, the prevalence of chronic diseases is high. Although the few studies on lifestyle interventions in rural areas have generally found favorable outcomes, studies that include rigorous designs and adequate sample sizes and produce generalizable results are needed.

## Figures and Tables

**Table 1:** Dietary intake and physical activity interventions in rural populations

Study	Design	Sample	Results	Limitatios
Befort et al. (2012)	Quasi-experimental one-arm (6-month) group phone-based weight control intervention addressing lifestyle modification and breast cancer risk	34 White women ages 46–74 years from three rural cancer centers in Kansas	Significant changes for weight (−12.5 ± 5.8 kg); waist circumference (−9.4 ± 6.3 cm); daily energy intake (−349 ± 550 kcal/day); fruits and vegetables (3.7 ± 4.3 servings/day); percent kcal from fat (−12.6 ± 8.6%); and physical activity (1235 ± 832 kcal/week (p<0.001) were observed.	Small sample size; lack of a control group; uncertain generalizability
Brown et al. (2011)	Pre-test, post-test control group design with two groups: experimental (diabetes self-management education or DSME with access to a nurse case manager or NCM vs. DSME only)	165 Mexican American men and women ages 35–70 years in Starr County, Texas	There were non-significant improvements in diet and physical activity among experimental and comparison cohorts. Regardless of study arm, compared to men, women had significant reductions in body mass index (BMI). The number of NCM contacts was proportional to DSME attendance.	Small sample size; uncertain generalizability
Carter et al. (2015)	Observational study (12-week) dietary intake, physical intervention, and hypertension	55 (39 female, 16 male) African Americans ages 35–75 years in Bullock County and Macon County, Alabama	Among Bullock county participants, body weight decreases ranged from 0.69 to 3.40%, with the exception of a dance group. In Macon County participants lost weight irrespective of the exercise regimen, with those involved in floor exercise losing the most weight (11.18%).	Small sample size, lack of a control group; uncertain generalizability
Fahs et al. (2013)	Multi-site randomized control trial (14 months) comparing 2 strategies (stage-matched nursing (SMN) and community intervention (CI) vs. CI alone) in changing CVD risk factors (diet, physical activity, and/or smoking)	117 non-Hispanic White (87.2%) Black (8.5%) and Hispanic (1.7%) women with a mean age of 50 years from 1 rural county in New York and 2 rural counties in Virginia	The SMN + CI cohort had significant increases in fruits and vegetables and reduced diastolic blood pressure; CI participants had significant reductions in cholesterol. For both groups, Framingham risk scores were reduced significantly post-intervention.	Small sample size, high attrition rate, uncertain generalizability; possible contamination across cohorts
Hageman et al. (2014)	Three-arm community-based clinical trial (12-month intervention with 12-month follow-up) comparing standard advice, intervention Internet or intervention print related to hypertension	289 women ages 40–49 years (mean age 56.4 years) primarily white (98%) from central Nebraska	Web-based and print-mailed groups improved more than standard advice group for waist circumference (p = 0.017 and p = 0.016, respectively); % daily calories from fat (p = 0.018 and p = 0.030) and saturated fat (p = 0.049 and p = 0.013); daily servings of fruit and vegetables (p = 0.008 and p < 0.005); and low fat dairy (p < 0.001 and p = 0.002).	Self-reported physical activity outcomes; possible contamination across cohorts
Hu et al. (2014)	Quasi-experimental family-based intervention (8-weeks) pilot study of diabetes self-management behaviors	26 Hispanic men (25%) and women (75%) average age 50 years and 37 family members, men (30%) and women (70%) average age 40.6 years in rural central North Carolina	Participants had higher levels of intake of healthy foods post-intervention. No significant changes in levels of physical activity were found among patients with diabetes or family members.	Small sample size, no control group; lack of randomization, uncertain generalizability
Kegler et al. (2012)	Quasi-experimental design coach-based (6-week) intervention (family goal setting and behavioral contracting to make home environments more supportive of healthy eating and physical activity) related to obesity	90 households with African American and White men and women 40–70 years and 18 years in Cook County and Randolph County, Georgia	Intervention households reported significant improvements in food inventories, purchasing of fruit and vegetables, healthier meal preparation, meals with the TV off, family support for healthy eating, increased exercise equipment, and family support for physical activity relative to comparison households. Intervention households also reported that the percent of fat intake decreased significantly, but there were no changes for fruit and vegetable intake, physical activity, or weight among intervention relative to comparison households.	Small sample size, lack of randomization, insufficient intervention period, less intensive intervention
Kim et al. (2008)	Quasi-experimental design with an intervention group and a delayed intervention control group	73 African Americans (71% female) mean age 54.1 years in rural North Carolina	Small groups led by trained community members met weekly for 8 weeks and emphasized healthy nutrition, physical activity, and faith’s connection to health. The mean weight loss in the intervention group was 3.60 lb, compared to 0.59 lb in the control group (P<0.001). The intervention was also associated with an increase in recreational physical activity (P<0.01). There was no significant difference in fruit and vegetable consumption.	Non-randomized design, small sample size, use of self-reported information, uncertain generalizability
Landry et al. (2015)	6-month, community-based, pre-post trial of an intervention consisting of motivational enhancement, social support, pedometer diary self-monitoring, and educational sessions	269 adults (94% African American, 85% female, mean age 44 yrs) in Hattiesburg, Mississippi	For the dietary and physical activity outcome variables, temporal changes were observed only for sugar intake and steps per day. Sugar intake decreased by about 3 teaspoons and physical activity increased by about 2,010 steps per day.	Lack of a randomized controlled design, uncertain generalizability, use of self-reported measures
Lilly et al. (2014)	Multi-site observational problem solving intervention to address barriers to lifestyle change	81 participants average age 52.8 years from 3 underserved populations: 28 Hispanic or non-Hispanic women in North Carolina, 31 African American women in West Virginia, and 22 adults in Appalachia	The intervention resulted in significant improvement in problem-solving skills (P < 0.001) and perceived stress (P < 0.05). Diet, physical activity, and weight remained stable, although 72% of individuals reported maintenance or increase in daily fruit and vegetable intake, and 67% reported maintenance or increase in daily physical activity.	Small sample size, no control group, insufficient intervention period, uncertain generalizability
Mayer-Davis et al. (2004)	Randomized-controlled clinical trail (12 months) with 3 arms: intensive intervention focused on Diabetes Prevention Program goals; reimbursable intervention and usual care controls	152 African American and non-Hispanic White men and women mean age 60 years in two rural counties in South Carolina	Participants in the intensive intervention lost more weight than those in the usual care cohort (2.6 kg vs. 0.4 kg, p<0.01); 12% of the intensive lifestyle participants gained at least 2 kg compared with 27% of the usual-care participants (from χ2 statistic, P < 0.05). There were no weight changes between the reimbursable and usual care participants.	Small sample size, uncertain generalizability
Parker et al. (2010)	Quasi-experimental (pre-post-test) 10-week intervention (spiritually based vs. non-spiritually based)	35 African American women (ages 25–64 years) in 3 rural South Carolina counties	Both interventions led to significant reductions in BMI but the spiritually based intervention (z = −1.97, P<0.01) led to greater reductions in BMI. For the spiritual group, statistically significant improvements were found in physical activity (*z* = −2.74, P<0.01)	Non-randomized design, small sample size; uncertain generalizability
Ries et al. (2014)	Quasi-experimental two-arm community-based intervention (6-months) focused on health information and goal setting support through group meetings and tailored newsletters	485 (208 intervention, 277 comparison) women (20% White, 63% Black, and 10% others) mean age 47.5 years in rural North Carolina	Intervention compared to comparison participants were more likely to move from contemplation to action/maintenance for the goals of improving diet (58% intervention, 44% comparison, p=0.04) and physical activity (56% intervention, 31% comparison, p≤0.0001).	Use of self-reported data
Robles et al. (2014)	Prospective cohort pilot (12-week) vigorous physical activity and nutrition education study	33 predominantly Hispanic (90%) overweight or obese girls (74%) and boys ages 8–11 years (mean age 9.6 years) in Bailey County, Texas	Positive survey results at 3 months indicated a decrease in fried/sweet foods; increase in exercise; decreases in video games and computer use; and a change in knowledge regarding the selection of the most healthy food group servings per day.	Small sample size, lack of a control group, short intervention duration
Scarinci et al. (2014)	Cluster randomized control trial (5-weeks) randomized to either healthy eating and physical activity or breast and cervical screening with 12 and 24-month follow-up	565 African American women, ages 45–65 years (mean age 53.9 years) randomized by counties in Alabama’s Black Belt (Dallas, Marengo, Sumter, Lowndes, Green, and Choctaw)	Participants in the healthy lifestyle arm (n=188) showed significant positive changes compared to the screening arm (n=121) at 12-month follow-up with regard to decrease in fried food consumption and an increase in fruit/vegetable intake (69%) and physical activity. At 24-month follow-up, these positive changes were maintained with healthy eating behaviors, but not engagement in physical activity.	Large detention differences across intervention arms; use of self-reported outcome measures
Tussing-Humphreys et al. (2013)	Quasi-experimental (6-month) for improving diet quality and increasing physical activity	403 African American men and women mean age 47 assigned to control (n=208) or intervention (n=195) in the Lower Mississippi Delta region	Diet quality components, including total fruit, total vegetables, and total quality improved significantly in both control (mean [standard deviation], 0.3 [1.8], 0.2 [1.1], and 3.4 [9.6], respectively) and intervention (0.6 [1.7], 0.3 [1.2], and 3.2 [9.7], respectively) groups, while significant increases in aerobic (22%) and strength/flexibility (24%) physical activity indicators were apparent only in the intervention group.	Self-reported diet and physical activity, lack of objective measure of physical activity, uncertain generalizability
Walker et al. (2009)	Randomized-controlled community-based clinical trial (12-month intervention period with 12 month follow-up) of computer-tailored newsletters (intervention) vs. generic newsletters (control)	225 non-Hispanic White and Hispanic women ages 50–69 years in two similar rural areas	From baseline to 6 months, there were significant increases in stretching and strengthening exercises, fruit and vegetable servings, and decreases in % calories from fat among intervention and control groups. From baseline to 12 months, intervention participants had greater increases in moderate activity, fruit and vegetable servings, and a reduction in % fat calories.	Self-reported measurement of dietary intake and physical activity; uncertain generalizability
Zoellner et al. (2013)	Randomized control pilot study (15-weeks) guided by CBPR principles, that provided 2/weekly access to group fitness classes with (Group 1) and without (Group 2) weekly nutrition and physical activity education	91 African American (62%) and White mostly female (91%) ages 18 years of age or older in the Dan River Region in south-central Virginia and north-central North Carolina	There were significant improvements in waist circumference (−1.4 inches), systolic blood pressure (−4.3 mmHg), and HDL-cholesterol (+7.9 mg/dL) (p<0.001). Self-reported walking per day was 44.8 (SD±52.2) minutes at enrollment and 65.9 (SD±89.7) minutes at 6 months (P=0.154).	Uncertain generalizability
